# Hydrogel Beads of Amidoximated Starch and Chitosan as Efficient Sorbents for Inorganic and Organic Compounds

**DOI:** 10.3390/gels8090549

**Published:** 2022-08-30

**Authors:** Diana Felicia Loghin, Melinda Maria Bazarghideanu, Silvia Vasiliu, Stefania Racovita, Marius-Mihai Zaharia, Tudor Vasiliu, Marcela Mihai

**Affiliations:** Petru Poni Institute of Macromolecular Chemistry, Aleea Grigore Ghica Voda 41A, 700487 Iasi, Romania

**Keywords:** grafted starch, ionotropic gelation, covalent cross-linking, molecular dynamics simulation, sorption capacity

## Abstract

The synthesis of hydrogel beads involving natural polymers is, nowadays, a leading research area. Among natural polymers, starch and chitosan represent two biomolecules with proof of efficiency and low economic impact in various utilization fields. Therefore, herein, the features of hydrogel beads obtained from chitosan and three sorts of starch (potato, wheat and rise starches), grafted with acrylonitrile and then amidoximated, were deeply investigated for their use as sorbents for heavy metal ions and dyes. The hydrogel beads were prepared by ionotropic gelation/covalent cross-linking of chitosan and functionalized starches. The chemical structure of the hydrogel beads was analyzed by FT-IR spectroscopy; their morphology was revealed by optical and scanning electron microscopies, while the influence of the starch functionalization strategies on the crystallinity changes was evaluated by X-ray diffraction. Molecular dynamics simulations were used to reveal the influence of the grafting reactions and grafted structure on the starch conformation in solution and their interactions with chitosan. The sorption capacity of the hydrogel beads was tested in batch experiments, as a function of the beads’ features (synthesis protocol, starch sort) and simulated polluted water, which included heavy metal ions (Cu^2+^, Co^2+^, Ni^2+^ and Zn^2+^) and small organic molecules (Direct Blue 15 and Congo red).

## 1. Introduction

In the last few years, extensive research has been undertaken to obtain specialized and selective sorbents containing natural polymers as a cheap and environmentally friendly solution for water cleaning. In this respect, polysaccharide-based hydrogels were studied, taking advantage of their low cost, availability, non-toxicity and biodegradability [1,2]. Among the polysaccharides, starch [3,4,5,6,7,8] and chitosan (CS) [9,10,11,12,13,14,15] have attracted the attention of the scientific community due to their physico-chemical characteristics, chemical stability, and excellent selectivity resulting from the presence of chemical reactive groups (hydroxyl, acetamido or amino functions) in polymeric chains. Moreover, these products are abundant, renewable, and biodegradable, and are able to physically and chemically bind to a wide range of molecules [16,17,18,19,20,21,22].

A number of studies have shown that polymers containing amidoxime groups have high complex-forming capabilities with metal ions and can be successfully used in metal ion removal from aqueous solutions [23,24,25,26,27,28,29,30]. Amidoxime hydrogel beads of modified alginate and amidoximated synthetic polymers have been successfully synthesized and used as sorbents for dyes, showing selective adsorption towards cationic dyes in the presence of anionic/cationic mixed dyes [31]. Usually, the synthesis of a sorbent with amidoxime groups involves the incorporation of a nitrile group into a polymer matrix, followed by the conversion of the nitrile group into an amidoxime group by treatment with an alkaline solution of hydroxylamine. For instance, sorbents containing amidoxime groups have been obtained by reacting acrylonitrile-divinyl benzene copolymer beads with hydroxylamine [32]. The introduction of amidoxime groups into acrylonitrile-grafted cellulose by interaction with hydroxylamine has also been investigated [33].

Starch is an interesting bio-material, due to its abundance and low cost, but has poor mechanical properties and the fact that it is highly hydrophilic. To overcome these drawbacks, chemical modification of starch is usually applied, mainly by grafting reactions. A large range of polymers can be grafted on starch by ring-opening and radical polymerizations of various monomers in order to modulate the properties of the final products [34]. For example, poly(amidoxime) ion exchange resins were synthesized from polacrylonitrile grafted sago starch, with their batch binding capacity for different metal ions being pH dependent [27,35]. In our previous studies, potato starch (PS) was grafted with acrylonitrile (AN) by the redox initiation by Ce^4+^ ions [36] and then the amidoximated (Ax) derivative was obtained [37]. Ionic composites based on crosslinked CS and amidoximated potato starch were also obtained and used as super-sorbents for copper ions, with reusability up to five sorption/desorption cycles, with no significant decrease in their sorption capacity [36]. Furthermore, the acrylonitryle grafted reaction, with the same Ce^4+^ ions as the initiator, was tested on three sorts of starch, namely PS, wheat (WS) and rice (RS) starches, followed by obtaining soluble derivatives by hydrolysis [5] The studies showed that the amylose/amylopectin content in starch and the grain size influenced the grafting performance, which reached 13.81%, 9.71% and 8.22% for PS, WS and RS, respectively [5].

Therefore, herein, the features of three sorts of starch (PS, WS and RS) grafted with acrylonitrile (PSgAN, WSgAN and RSgAN) and then amidoximated (PSgAx, WSgAx and RSgAx) were deeply investigated, following the formation of composite hydrogel beads with CS. The current study starts with the premise that the use of starch from different botanical sources in grafting reactions can influence the properties of the obtained materials, and consequently their properties. Thus, we aimed to prepare hydrogel beads by ionotropic gelation and covalent cross-linking of CS and functionalized starches as SgAN or SgAx. The chemical structure of functionalized starches and the obtained hydrogel beads was followed by FT-IR spectroscopy; their morphology was revealed by optical and scanning electron microscopy, while the samples’ crystallinity changes in relation to the functionalization strategies was evaluated by X-ray diffraction. Molecular dynamics simulations were used to determine the influence of the grafting reactions and grafted structure on the starch conformation in solution and their interactions with chitosan. The sorption capacity of the beads for Cu^2+^, Co^2+^, Ni^2+^ and Zn^2+^ ions and for Direct Blue 15 and Congo red dyes was followed in batch experiments, as a function of the beads’ features (synthesis protocol, starch sort), and contact time.

## 2. Results and Discussion

### 2.1. Starch Functionalization

**Figure 1:**
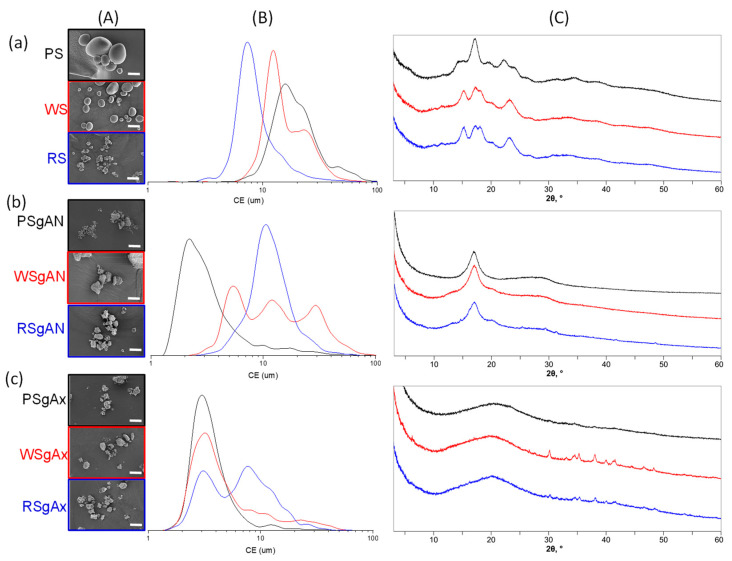
SEM images (scale bar 20 µm) (**A**), circular equivalent (CE) diameter (**B**) and X-ray diffractograms (**C**) of initial (**a**) and grafted AN (**b**) and Ax (**c**) starches.

Previous studies showed that the grafting reaction, in the current applied conditions, took place on the amylose part of starch [36] and the amylose contents of the used starch were as follows: 20–21% in PS, 23–30% in WS and 17–30% in RS [5]. Herein, the three types of starch were functionalized by grafting acrylonitrile to the amylose component of starch (SgAN samples), followed by amidoxime transformation (SgAx samples) (see details in Section 4.2). SEM images (Figure 1(aA)) revealed that starch granules have different morphologies. PS has a spherical, elliptical or irregular shape, whereas WS is predominantly spherical shaped, both with a smooth surface; RS particles show polyhedron-type shapes with sharp edges. Figure 1(bA,cA) show that PS and WS granules after AN grafting (SgAN), and also after amidoximation (SgAx), had irregular shapes and sizes, most of them being smaller than the initial granules, whereas RS-based samples show small changes in morphology, mainly with the loss of sharp edges.

The particle size analysis of native starches (Figure 1(aB)) shows that PS has the largest granule size (mean diameter, Dm = 15.58 µm), with WS and RS being characterized by lower particle sizes (Dm = 10.1 µm and 8.7 µm) and similar particle size distribution (with a shoulder for larger sizes suggesting particle aggregation), which was almost unimodal for each starch type. After AN grafting, the size distribution (Figure 1(bB)) undergoes SEM observation (Figure 1(bA)), and shows that the highest grain fragmentation is observed for the PSgAN and WSgAN, with smaller particle aggregation being found for RSgAN. After amidoximation, the samples show a population of particles with a mean diameter of about 3 µm (Figure 1(cB)) and the aggregation is characteristic also for the RS grains. Moreover, the size and morphology modifications observed in Figure 1A,B can be explained by the selectivity of the grafting and amidoximation reactions to the amylose part of the starch grains and the amylopectin part was partially removed during the starch grain functionalization process [36]. Thus, during the gelatinization process, which took place in the first reaction step, the amorphous amylose part of the grains becomes available to the Ce^4+^ ions, which interacts with the -OH groups located at the C2 and C3 carbon atoms.

The powder XRD diffraction analyses of PS, WS and RS and grafted starch are presented in Figure 1C. Generally, the X-ray diffraction results of starch beads are classified as A, B or C type, and depend upon the double-helical amylopectin chain arrangement. The A type pattern is a result of close-packed arrangements with a water molecule connection between the double helix, the B type is open hexagonal packing with water in the central cavity and type C is quite similar to the A pattern, except for the appearance of the peak around 5° [38]. The X-ray diffractogram of PS revealed a typical B-type pattern [39], with a strong reflection peak (100) at around 17°, relatively low intensity peaks at around 5° and 22° and shoulders around 15° and 24° (2θ). The diffractograms of WS and RS were practically identical, with the strongest peaks at approximatively 15°, 17° and 23° (2θ). The A-type pattern of WS and RS can be proved by the appearance of the shoulder at around 18° (2θ), a signal that is characteristic to this starch type. After being grafted with AN (Figure 1(bC)), the characteristic diffraction peaks of native starch disappeared in the XRD patterns with the appearance of a new intense peak around 17° (2θ), which can be ascribed to the structure modification following the AN grafting. As compared to native and AN-grafted starch, the XRD spectra of SgAx displayed a typical V-type crystalline structure [40] with a wide peak at around 20° (2θ). This wide peak can be attributed to the hydroxylamine functionalization reactions, which lead to the partially destroyed crystallinity of starch. The XRD results also confirmed our previous studies [36], where it was shown by ^1^H-NMR studies that the grafting reaction takes place mainly at the amylose component of starch, the amorphous component of starch molecules.

**Figure 2:**
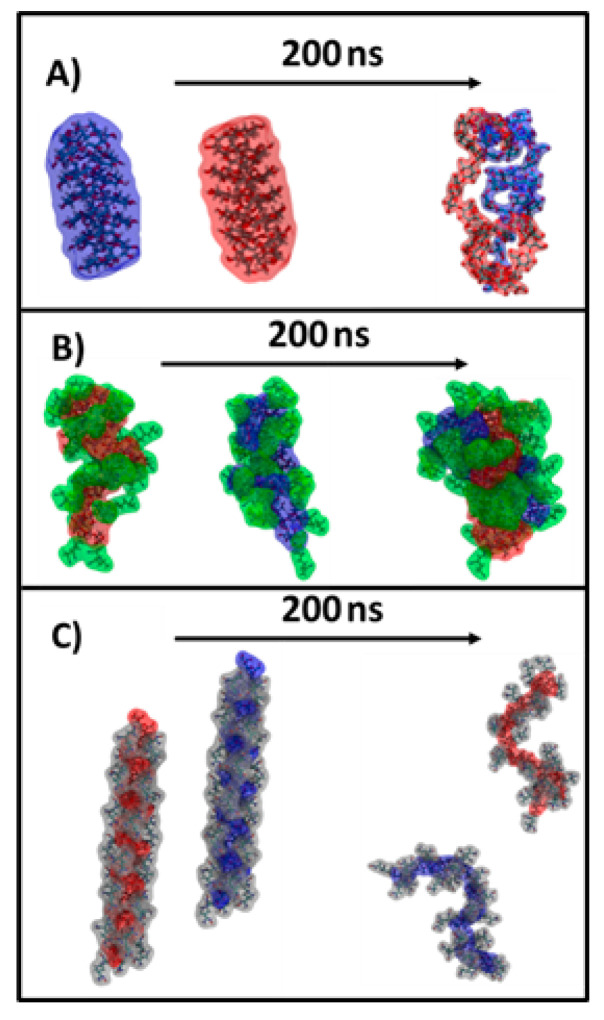
Snapshots depicting the starting and final structure of the 3 simulated systems: (**A**) amylose from starch, (**B**) SgAN and (**C**) SgAx. The starch molecules are colored in red and blue, the AN side chains are colored in green and the Ax side chains are colored in silver. Water has been omitted for clarity.

Molecular dynamics (MD) simulations were performed in order to visualize the conformation modifications of the starch (amylose) backbone after AN grafting reaction and its further amidoxime functionalization. The different types of patterns observed in starch crystallization are determined mainly by the interaction between two starch molecules. This is why the three simulated systems contained two identical starch molecules (amylose, SgAN and SgAx) solvated in water (see details in Section 4.5). Figure 2 depicts the initial conformation of the system and the final structure obtained after 200 ns of simulation.

The amylose starch molecules start with separated conformation, with a distance between the two molecules of >20 Å, to eliminate any bias in the interaction that could take place. After 200 ns, it can be observed in Figure 2A,B that the unfunctionalized and the SgAN molecules are interacting with each other, while the SgAx molecules remain separate. The unfunctionalized starch molecules are wrapped around each other in a way that is similar to a double-helical structure (Figure 2A). Similar observations were found in the literature [41], which also showed that this association could have two main crystal forms, A and B, as already observed for our starch samples in Figure 1C. The AN grafted starch behaves in a similar manner to the previous system, with the main starch chains wrapped around each other and the side chains exposed to the exterior (Figure 2B). The Ax functionalized starch behaves differently to the other investigated starch, in that the molecules remain isolated for the entire simulation (Figure 2C). The structures obtained in the MD simulation corroborate well with the XRD result (Figure 1C). In detail, in the case of the unfunctionalized starch molecules, the amylopectin crystalline part of the starch, and also the amylose part, can interact with each other and assemble in structures that are precursors to the A or B type patterns. In the case of the SgAN, although the amylose main chains interact with each other in a similar way to the previous system, the side chains located at the exterior and the absence of amylopectin leads to a decrease in crystallinity. Thus, the reflection peak (100) at around 17° observed in all SgAN samples (Figure 1(bC)) can be ascribed to the remaining organization of the starch main chains while the AN side chains determine a loose structure. In the case of SgAx, the MD simulation clearly depicts the V-type pattern of single starch molecules in an extended conformation.

### 2.2. Hydrogel Beads of Functionalized Starch and Chitosan

**Figure 3:**
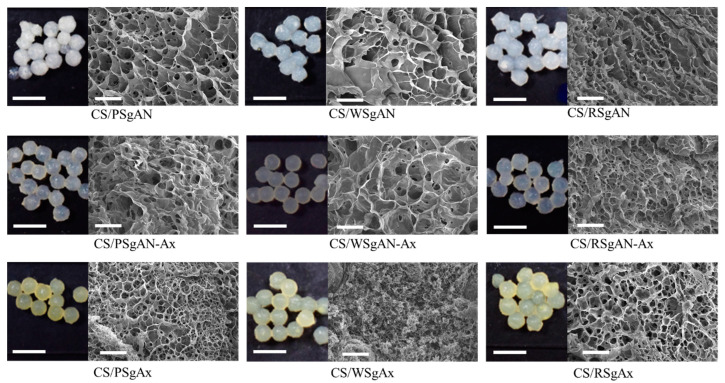
Optical (scale bar 5 mm) and SEM (scale bar 20 µm) images of composite beads.

The composite hydrogel beads formation follows two routes, which include using SgAN or SgAx as precursors and obtaining three types of composite beads, CS/SgAN, Cs/SgAN-Ax and Cs/SgAx (see details in Section 4.3). The beads are sphere shaped, as observed in the optical images of wet beads, and are porous, as shown by the SEM images of the surface of lyophilized beads (Figure 3).

The optical images in Figure 3 show some color changes as a function of the composite bead synthesis pathway: the beads obtained with SgAx keep the yellow color of amidoximated starches, whereas after post-amidoximation translucid beads were observed, with a slight yellow tinge. In addition, the beads’ size in swelled form is not influenced by the synthesis pathway, as is the surface morphology observed in the SEM images. Thus, CS/SgAN beads have uniform pore distribution at the beads’ surface, with thin walls. After bead amidoximation (CS/SgAN-Ax samples), the porosity slowly decreased and the pore walls becomes thicker. A different morphology of the composite beads was obtained when SgAx was employed in the bead formation process, i.e., a smaller size of the pores was obtained for all the investigated samples, most probably since the -NH_2_ groups in SgAx could also be crosslinked by epichlorohydrin (ECH), as is the case for the similar groups in CS; thus, a double crosslinked network is formed.

The samples’ porosity in the swollen state was estimated by measuring 50 pores in each SEM image from Figure 3; the resulted mean values of area, perimeter, aspect ratio (ratio of major/minor axis of pores) and Feret diameter (the longest distance between any two points along the selection boundary) are included in Table 1.

**Figure 4:**
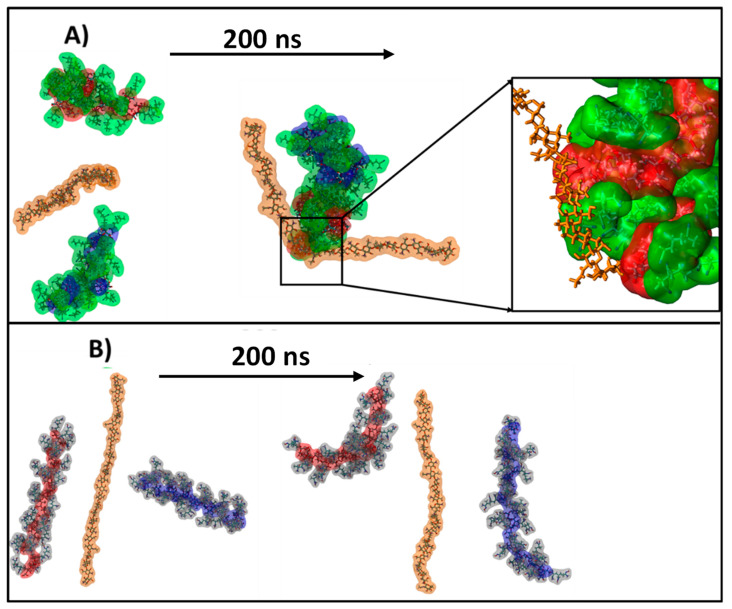
Snapshots depicting the starting and final structure of the simulated systems containing CS and (**A**) SgAN and (**B**) SgAx. The starch molecules are colored in red and blue, the AN side chains are colored in green, the Ax side chains are colored in silver and the chitosan is colored in orange. Water has been omitted for clarity.

Thus, the obtained values of the aspect ratio are higher than 1.00 (circles), meaning that the pores have elongated shapes. In addition, the Feret diameter values showed that the larger (the higher diameter) and the more irregular shaped (with higher aspect ratio values) pores were obtained when WS was used in hydrogel bead synthesis and were smaller with RS. The same trend was observed after bead amidoximation (SgAN-Ax samples). Larger amylose contents in RS (up to 30%) favor better grafting reactions and resulted in its better embedment into the hydrogel beads, leading to smaller pore sizes. PS and WS, with almost the same amylose content (up to 20 and 23–30%, respectively) formed hydrogel beads with larger pores. The amidoximated starch directly used in bead formation led to smaller size pores, as the starch had a very small influence on their porosity. In this synthesis route, the SgAx participation to crosslinking along with CS, and also the elongated form of SgAx both contributed to obtaining homogeneous beads with very close pore sizes, irrespective of the starch source. This observation is also supported by the MD simulations of the Ax-grafted starch that showed the side chains extended in the solvent, which were completely free to interact with ECH (Figure 2). To further support this observation, MD simulations of two systems containing two starch molecules of SgAN or SgAx and one CS molecule were performed (Figure 4).

The MD simulations showed that CS is able to interact with the SgAN molecules, coming into contact with both the AN side chains and the amylose backbone (detail of Figure 4A), while in the case of SgAx molecules, no contact between the CS an SgAx can be observed for the entire simulation (Figure 4B). This is due to the fact that both molecule types have a positive charge and repel each other.

The hydrogel beads’ elemental composition was followed by EDAX analysis and Table 2 shows the obtained results both at their surface and in the sections. The values were compared with the values calculated taking into account the ratio between the grafted starch in the synthesis process and CS (1:4) and ECH (1:0.37), considering, for functionalized starches, the mean grafting of three units to each monomer unit.

Thus, the C/N atomic ratios are higher than the calculated values both at the surface and inside the beads. The lower N content in the beads can be ascribed to a synergy of factors, mainly because of the lower contribution of the AN-grafted side chains in starch and/or a higher contribution of the CS chains inside the beads. By considering the small length of the grafting chains, we may assume that the grafted chains can be hindered by the macromolecule conformation and, as observed in the MD simulation, with the SgAx behaving as elongated macromolecules, with easily accessible functional groups. This is also related to the contribution of CS to the formation of the beads, with the partial crosslinking of SgAx decreasing the amount of ECH available to crosslink the CS chains and the free chains being most probably partially removed during the final washing steps. The above-mentioned observations are sustained by the C/O atomic ratios, which are higher than the calculated ones. The comparison of the C/N and C/O atomic ratios at the surface and inside the beads revealed that there are some differences in the elemental composition and that inside the beads, the atomic ratio values are smaller than outside but are the closest to the calculated values.

**Figure 5:**
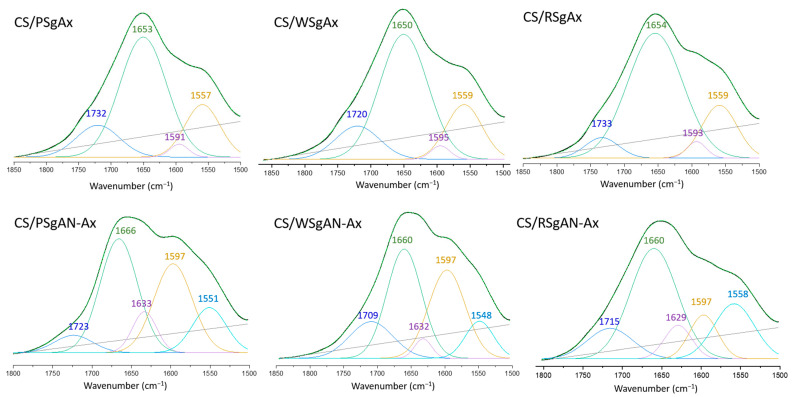
Deconvolution of 1800–1500 cm^−1^ region of FTIR spectra of CS/amidoximated starch beads obtained by the two procedures.

Further proof of the samples’ chemical structure was obtained by FTIR and is shown in Figures S1–S3 (supplementary information), where the characteristic peak of AN is observed just in the CS/SgAN samples and the characteristic peaks for starch and CS are found in all the spectra. Nevertheless, as shown in previous studies [36], the formation of amidoxime can be demonstrated in FTIR at about 1650 cm^−1^ or 933 cm^−1^, assigned to the C=N and N-O bonds in oximes, respectively. Therefore, the 1800–1500 cm^−1^ area was selected and the peak deconvolution was performed for both types of amidoximated beads, CS/SgAN-Ax and CS/SgAX (Figure 5, Table S1).

Thus, the region 1800–1500 cm^−1^ of the three spectra of the CS/SgAx samples is deconvoluted in four individual peaks, irrespective of starch nature, which can be ascribed as follows: 1733–1720 cm^−1^ to carbonyl groups C=O, resulting from the grafting reaction and anhydro glucose ring opening, 1654–1650 cm^−1^ to C=N in the oxime groups (the highest contribution), 1591–1595 cm^−1^ to -NH_2_ groups on both CS and modified starch (with the smaller contribution) and 1557–1559 cm^−1^ to vibrations of the protonated amine group. The same region in the FTIR spectra of the composite beads obtained by the amidoximation of the already formed CS/SgAN beads (namely CS/SgAN-Ax samples) demonstrated five individual peaks, with the peak ascribed to C=N in the oxime groups being shifted to about 1660 cm^−1^ and the new peak at about 1630 cm^−1^ being ascribed to the -OH groups bending. Furthermore, for these samples, the contribution of the peak at 1660 cm^−1^ diminished and the contribution of that at about 1597 cm^−1^ increased, suggesting a higher amount of free amino groups obtained by the post bead formation amidoximation reaction groups, which are evidently not involved in the crosslinking reactions with ECH.

**Figure 6:**
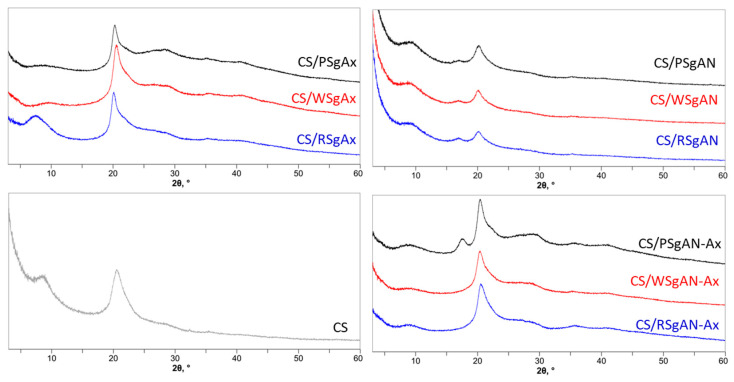
X-ray diffractograms of CS/SgAx, SgAN and SgAN-AX composite beads as compared to bare CS.

The modifications in the starch/CS crystallinity after hydrogel bead synthesis was followed by X-ray diffraction (Figure 6).

The chitosan sample shows diffraction peaks at about 9° and 20° (2θ), which could be found in all the samples after hydrogel bead formation, as shown in Figure 6. In addition, the diffraction peak at 17° (2θ), characteristic for grafted starch (Figure 1(bC,cC)), was observed in the CS/SgAN samples and was also found after their amidoximation for beads obtained with PSgAN. For the samples obtained with the other AN-grafted starch (RS and WS) and that with SgAx, the peak at 17° (2θ) was not observed, with the peak at about 9° being evident mainly in CS/RSgAx beads. To conclude, the X-ray diffraction analysis showed that the samples prepared by the two methods, either using SgAN and beads post amidoximation or by using SgAx, still contain the functionalized starch structures, suggesting good incorporation of functionalized starch dispersion into the beads.

### 2.3. Swelling Behavior of Hydrogel Beads

**Figure 7:**
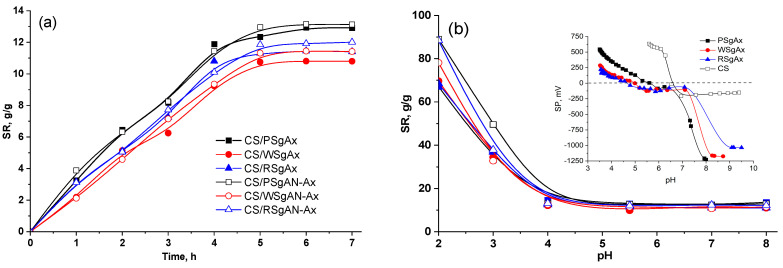
Swelling of the composite hydrogel beads as a function of time at pH = 6 (**a**) and different pH values (**b**); inset in (**b**) potentiometric titration curves of SgAx and CS.

The swelling behavior represents an important characteristic of a sorbent in its capacity to retain different ions or small molecules. Therefore, the swelling degree was evaluated as a function of time and pH (Figure 7). As shown in Figure 7a, in distilled water with a pH of about 6, the swelling equilibrium was reached in almost five hours for all the beads, irrespective of the sort of starch or the method applied in bead synthesis. Furthermore, there were almost no differences between the swelling degree values for the beads prepared with SgAx compared to those obtained with SgAN and those amidoximated. There are small differences between the swelling of the samples prepared with different sorts of starch, which can be ascribed to the differences in the starch grain sizes (Figure 1), with the largest PS grains resulting in a larger swelling capacity, as compared to the other starch sorts. Nevertheless, the swelling is also connected to the amylose/amylopectin ratio, which is almost 20/80 in PS, 23/77 in WS and 30/70 in RS [5]. Thus, for the lower grain size of WS and RS, the larger amylose content in RS results in a larger swelling capacity compared to that of the WS-based hydrogel beads.

Almost similar and constant values were obtained when the pH of the swelling media varied between 4 and 8 (Figure 7b), irrespective of the sort of the starch and the bead preparation procedure. As shown in the inset in Figure 7b, the point of zero charges of CS is located at a pH of about 6.5; below this pH value, in the presence of hydronium ions, the primary amino groups (–NH_2_) of chitosan can be protonated (–NH_3_^+^). The amidoximated starches show different points of zero charges, for example, at 4.6, 5 and 5.7 for RSgAx, WSgAx and PSgAx, respectively. Before these values, the primary amino groups of amidoximated starch and CS are protonated; therefore, below pH 4, the interpolymeric electrostatic repulsions in the composite beads and also the intramolecular repulsions between the ionized groups lead to an increase in the bead size; consequently, the equilibrium swelling ratio values increase with the pH decrease (swell between 70 and 90% at pH 2). On the contrary, at pH values up to the CS isoelectric point (6.5), ionization of the -OH groups in the amidoxime functional groups occurs, which could be associated with the protonated amino groups, decreasing the beads’ swelling capacity. Furthermore, the swelled beads are stable across the whole range of the tested pHs, suggesting that the crosslinking process was effective.

### 2.4. Sorption of Metal Ions by Hydrogel Beads

**Figure 8:**
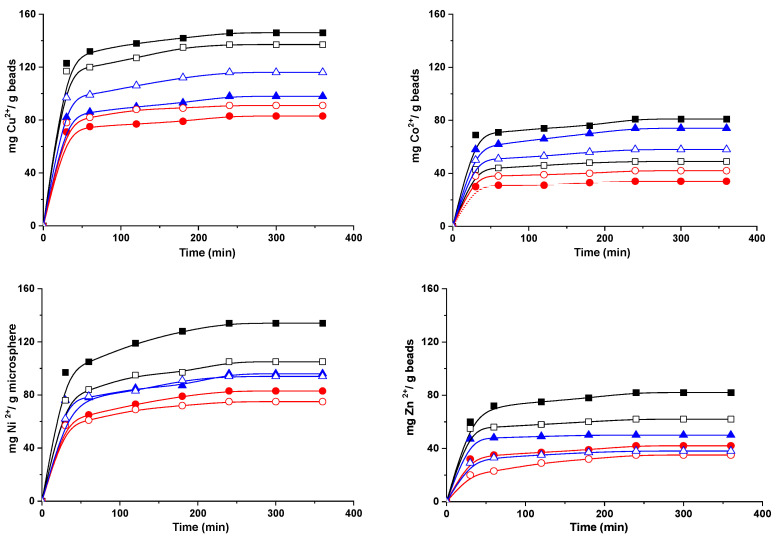
Sorption of Cu^2+^, Co^2+^, Ni^2+^ and Zn^2+^ onto composite beads based on PS (square), WS (circle) and RS (triangle) and using hydrogel beads CS/SgAx (close symbols) or CS/SgAN-Ax (open symbols).

Composite hydrogel beads with amidoximated groups (CS/SgAN-Ax and CS/SgAx) were tested as supports for the uptake of Cu^2+^, Co^2+^, Ni^2+^ and Zn^2+^ ions from aqueous solutions, in batch experiments. The influence of contact time on the hydrogel beads’ sorption capacity for metal ions is represented graphically in Figure 8, and shows that the time required to reach the equilibrium was about four hours for all the studied metal ions. For each metal ion, the hydrogel beads’ sorption capacity was influenced by the nature of the starch used to obtain the beads and the manner in which the beads were obtained. Nevertheless, the best sorption capacity for the investigated ions was obtained when CS/PSgAx hydrogel beads were used, with the highest affinity to Cu^2+^. Furthermore, with some exceptions, the beads obtained with SgAx show better sorption capacities for the investigated ions, as compared to the beads post-amidoximation, following the same trend as that found for the beads’ swelling vs. time (Figure 7a). For each sorbent, the beads’ affinity for the tested metal ions follows the same order, which is as follows: Cu^2+^ > Ni^2+^ > Zn^2+^ ≈ Co^2+^.

The sorption capacity is usually influenced by the ions’ properties, such as ionic radii, hydrated radius, atomic weight, electronegativity, and others, as already observed in other studies [42,43,44,45]. In this study, the ionic radius (Pauling) (Co 0.745 Å, Zn 0.74 Å, Cu 0.73 Å, and Ni 0.69 Å) and hydrated ionic radius (Zn 4.30 Å, Co 4.23 Å, Cu 4.19 Å, and Ni 4.04 Å) [46] represent the influence parameters. Furthermore, as shown in a previous study [47], the ions’ coordination with nitrogen ligands is usually arranged in preferential structures, with the octahedral structure being preferred by Co^2+^ and Ni^2+^, tetrahedral by Zn^2+^ and square planar by Cu^2+^. Nevertheless, the found affinity for ion sorption in noncompetitive conditions towards the amidoximated starch-based beads of Cu^2+^ > Ni^2+^ > Zn^2+^ ≈ Co^2+^ suggests that most probably, sorption is favored by the lower ionic radius and by the structural arrangements that allow for the smallest energy consumption, with copper ions best fulfilling these conditions.

**Figure 9:**
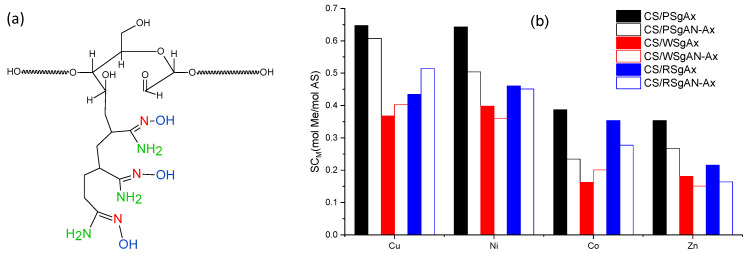
(**a**) Schematical representation of the three active sites (colored) of amidoxime functional group and (**b**) sorption capacity of hydrogel beads CS/SgAN-Ax or CS/SgAx expressed as moles of metal (Me)/moles of active sites (AS) in hydrogel beads.

Another important parameter is the initial concentration, which herein was set to 300 mg/L and corresponds to different molar concentrations, i.e., Zn 4.587 mmol/L, Co 5.093 mmol/L, Cu 4.724 mmol/L, and Ni 5.11 mmol/L. Thus, the molar sorption capacity of the gel beads was calculated as the moles of metal ions sorbed after 360 min per moles of active sites in the hydrogel (Figure 9). Each amylose unit is grafted with a mean of three amidoxime groups and each group has three active sites (Figure 9a) and each chitosan deacetylated unit has one active site (primary amino group) to interact with the metal ions. Thus, taking into account the amount of each component used in hydrogel bead preparation (see Section 4.3) and the number of active sites of each component, the calculated amount of active sites to interact with metal ions per gram of gels was found to be 3.55 mmol/g. As shown in Figure 9b, PSgAx-based hydrogel beads have the highest sorption capacity for all the investigated ions and their higher swelling degree most probably allowed the ions to easily reach the active sites. The similar beads obtained with RS and WS followed the same trend as the swelling degree, assuming a sorption process controlled by diffusion. Nevertheless, even if CS/PSgAN-Ax beads have similar swelling capacity to that of the CS/PSgAx beads (Figure 7), their sorption capacity is lower, irrespective of the sorbed metal ion, suggesting lower active sites are available for the coordination of the metal ions. The hydrogel beads prepared with the other two sorts of starch (RS and WS) show different behavior for each tested ion and as a function of the bead preparation procedure (using SgAx or post-amidoximated beads). Therefore, the complex sorption process as a function of the beads’ features, as well as the metal ions’ characteristics, should be carefully and deeper investigated.

### 2.5. Sorption of Dyes

**Figure 10:**
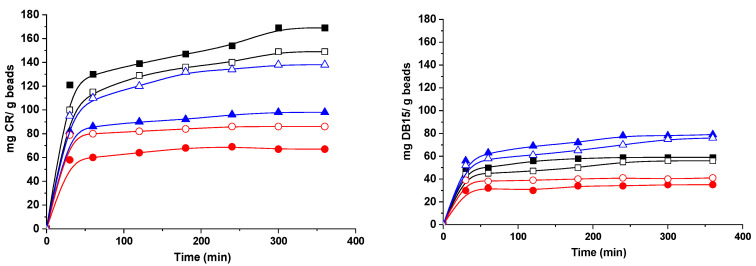
Sorption of dyes onto composite beads based on PS (square), WS (circle) and RS (triangle) and using CS/SgAx (close symbols) or CS/SgAN-Ax (open symbols) hydrogel beads.

Organic dyes are among the major concerns of water pollutants. Congo red (CR) and Direct Blue-15 (DB15) are organic dyes that can be easily dissolved in water, causing difficulties in their removal from contaminated water. Furthermore, they are toxic and carcinogenic, causing various diseases. Both dyes are secondary diazo dyes, with complex aromatic structures that make them non-biodegradable and quite stable. Moreover, they contain anionic sulphonic groups that can electrostatically interact with the protonated amino groups in the composite hydrogel beads. The sorption capacity of the hydrogel beads with amidoxime groups for both CR and DB15 is represented in Figure 10. The time required for dye adsorption was about six hours, as can be observed in the Figure 10, with higher values being obtained when CR was sorbed as compared to DB15. These differences are influenced by several factors, such as the origin of the starch, the bead synthesis method and the dyes’ characteristics.

Thus, PS-based hydrogel beads show the best affinity to CR, whereas for DB1, better results were obtained with RS-based hydrogel beads and lower values were found when hydrogel beads with WS functionalized starch were used, irrespective of the dye sorbed. Furthermore, better results were obtained with CS/SgAx than with CS/SgAN-Ax. This correlates with the chemical structure of the hydrogel beads and the amino groups of both CS and amidoximated starch could interact, in specific conditions, with the negatively charged dye molecules. The influence of the presence of ionic groups on the hydrogel matrix and their interaction with ionizable groups of dyes was also observed in other studies [48] The size of the dye molecules could also influence the hydrogels’ sorption capacities, with CR having the molar mass of 696.665 g/mol and DB15 of 992.80 g/mol, and is in direct relation to the hydrogel beads’ porosity (Figure 3). Thus, CS/PSgAx and CS/RSgAx show higher pore sizes as compared to CS/WSgAx; therefore, the smaller CR molecules could be sorbed inside them, whereas the sorption of DB15 with larger molecules was hindered.

## 3. Conclusions

In this study, the features of three sorts of starch (PS, WS and RS) grafted with acrylonitrile and then amidoximated were deeply investigated, following the formation of composite hydrogel beads with CS, and tested as sorbents for four heavy metal ions (Cu^2+^, Co^2+^, Ni^2+^ and Zn^2+^) and two dyes (DB15 and CR). The MD simulations show that the AN-functionalized starch behaves in a similar manner to the native one, as the main starch chains wrapped around each other and the side chains were exposed to the exterior, whereas the Ax-functionalized starch molecules remained isolated for the entire simulation. The structures obtained in the MD simulation corroborate well with the XRD result; in the case of the SgAN, the side chains located at the exterior of the wrapped arrangement and the absence of amylopectin leads to a decrease in crystallinity, whereas for SgAx, the V-type pattern of single starch molecules in a coiled conformation is found. The comparison of the C/N and C/O atomic ratios at the surface and inside the beads revealed that there are some differences in the elemental composition and that inside the beads, the atomic ratios values are smaller than outside, but are the closest to the calculated values. FTIR spectra of the CS/SgAx samples were deconvoluted in four individual peaks, irrespective of starch nature, which can be ascribed to both components’ functional groups, since the CS/SgAN beads post-amidoximation shows five individual peaks. The new peak and also the variation in the others’ intensity suggested a high amount of free amino groups obtained by the post bead formation amidoximation reaction, groups which are obviously not involved in any crosslinked reactions with ECH. The hydrogel beads show good sorption capacities for Cu^2+^, Co^2+^, Ni^2+^ and Zn^2+^ ions and for Direct Blue 15 and Congo red dyes, with their performances being influenced by the synthesis protocol and starch sort. Future studies must continue with other sorption-related experiments as a function of different parameters (pH, concentration, temperature) to elucidate the sorption mechanism for both inorganic and organic molecules.

## 4. Materials and Methods

### 4.1. Materials

CS powder, from Sigma-Aldrich, was used as received. The degree of acetylation of 15% and the average molar mass of 385 kDa were determined by the methods previously reported [49]. PS (humidity content < 10%, ash < 0.5%) and WS (humidity content < 15%, ash < 0.5%) were purchased from Fluka and were used as received. RS, epichlorohydrin (ECH), sodium hydroxide, methanol p.a., hydroxylamine chlorohydrate, metal ion salts (CoSO_4_·7H_2_O; NiSO_4_·6H_2_O; ZnSO_4_·7H_2_O and CuSO_4_·5H_2_O), Direct Blue 15 (DB15) and Congo red (CR) were purchased from Sigma-Aldrich and were used as received. Acrylonitrile was distilled at about 77 °C and kept at a low temperature.

### 4.2. Starch Functionalization

The starches grafted with acrylonitrile (SgAN), obtained using potato (PSgAN), wheat (WSgAN), or rise (WSgAN) starch, were synthesized and characterized as described in [5], using Ce(SO_4_)_2_ as the initiator in H_2_SO_4_ 0.4 M, at 27 °C, under stirring for 1 h, and then separated in methanol. The polyamidoxime-grafted starch (SgAx) samples were obtained by analogous reactions of nitrile groups of SgAN, using hydroxylamine in an alkaline medium [36]. SgAN was introduced in a two-necked flask, which was equipped with a stirrer and condenser placed in a thermostatic water bath. Then, the hydroxylamine solution was added to the flask, and the reaction was carried out under stirring for 5 h at 70 °C, and then for 24 h at room temperature without stirring. After completion of the reaction, the SgAx was separated from the solution by filtration and washed intensively with methanol:water (80:20, *v*/*v*) solution. The same procedure was applied for all the types of starch. The AN average grafted length of three AN/starch structural units was determined from the ^1^H-NMR spectra of SgAN [5] as the ratio of the integral AN characteristic peak at 3.1 ppm (ascribed to protons from CH groups) and starch characteristic peak located at 3.65 ppm (attributed to hydrogen at C2, C3 and C5 atoms).

### 4.3. Hydrogel Bead Synthesis

The composite beads were obtained by two similar methods that have already been published [36], using CS and amidoxime or acrylonitrile-grafted potato, wheat and rise starches, obtaining CS/SgAN and CS/SgAx beads. Shortly after, 0.5 g of SgAx or SgAN were first dispersed in 100 mL solution of CS 2% (*w*/*v*), and then 2 mL ECH was added as the crosslinker. The obtained mixture was dripped (through a syringe pump, ISPLab02) into an aqueous solution of sodium tripolyphosphate of 0.05 M, at room temperature, under gentle stirring (100 rpm). After 4 h, the formed beads were separated and transferred to an aqueous solution of 400 mL 0.1 M NaOH for 2 h, under slow stirring.

The beads prepared using SgAN were subjected to amidoximation by analogous reactions, obtaining CS/SgAN-Ax beads; the CS/SgAN beads and 70 mL hydroxylamine solution were introduced into a two-necked flask equipped with stirrer and condenser, and the reaction was carried out under mild stirring for 5 h at 70 °C. Finally, the prepared microspheres (obtained by both methods) were washed with distilled water at a neutral pH, and dried at 104 °C (Moisture Analyzers Precisa XM 60-HR, Precisa Gravimetrics AG, Dietikon, Switzerland).

### 4.4. Characterization Methods

Information on the surface morphology was evaluated using the Various G4 UC scanning electron microscope (Thermo Scientific, Brno-Černovice, Czech Republic), whereas the elemental composition with an energy dispersive X-ray spectroscopy analyzer (Octane Elect Super SDD detector, Ametek, Mahwah, NJ, USA) were analyzed by SEM. The investigations were carried out on samples sputtered with 10 nm platinum (Leica EM ACE200 Sputter, Leica, Wetzlar, Germany) in a high vacuum mode, using secondary electrons (Everhart-Thornley detector, FEI Company, Brno, Czech Republic) and concentric backscattered detectors. The samples’ porosity in the swollen state was estimated by selecting 50 pores in each SEM image using ImageJ 1.48v analyzing software (LOCI, University of Wisconsin-Madison, Madison, WI, USA) [50], measuring the area, perimeter, aspect ratio and Feret diameter of each one.

Optical images were obtained with a Nikon D3300 HDSLR camera, with an AF-P DX NIKKOR 18–55 mm f/3.5–5.6 G VR lens.

FTIR analysis of the grafted samples was performed using a Bruker FT-IR spectrometer. Each spectrum was scanned in the range 400–4000 cm^−1^, using 45 scans with a resolution of 4 cm^−1^ by the KBr pellet technique, using 2 mg of each sample. The spectra deconvolution was carried out using the Opus 4.7v software (Universität Stuttgart, Stuttgart, Germany).

The native starch, grafted starched (with AN and Ax) and CS-based composite beads were examined by X-ray diffraction by a Rigaku Miniflex 600 diffractometer (Rigaku, Tokyo, Japan), using CuKα-emissions in the angular range 3–60° (2θ), with a scanning step of 0.01° and a recording rate of 2°/min.

The diameter (circular equivalent) of native and AN and Ax-grafted starched was determined by a Morphologi G3SE particle characterization system (Malvern Instruments, Malvern, UK). The samples were carefully spread out over a glass plate. To evaluate the hydrogel beads’ diameter, only full shaped particles, which were non-aggregated, were measured.

Potentiometric titrations were carried out with a particle charge detector (PCD 03, Mütek, Germany). The pH variation between 3 and 10 was achieved using 0.1 and 0.01 M solutions of NaOH and HCl, respectively. The potential measurements were carried out using 1 mg beads in 10 mL Millipore water, at room temperature.

### 4.5. Molecular Dynamics Simulation

Three systems containing two starch molecules solvated in water and two systems containing two starch molecules and one chitosan molecule were built using the AmberTools 18 software (University of California, San Francisco, CA, USA) [51]. Each starch molecule contained 26 repetitive units of linear starch (amylose), and the grafted starch had three units of acrylonitrile or amidoxime grafted to each starch monomer. The chitosan molecule had 26 chitosan monomer units with 15% acetylation. The partial atomic charges were obtained by RESP using the R.E.D.-III.5 tools software [52]. The MD simulations were performed using the GAFF2 forcefield [53] for the starch molecules, the GLYCAM forcefield for the chitosan and the TIP3P water model [54] for the solvent and ions. GROMACS 2020 [55] was used to run the simulation with a temperature set at 300 K, by using V-rescale temperature coupling with a time constant of 0.5 ps. The pressure was controlled by the Parrinello–Rahman barostat and isotropic pressure coupling, with a constant time of 2.0 ps and compressibility of 4.5 × 10^−5^. Each simulation had a length of 200 ns, the composition of simulated systems being introduced in Table 3.

### 4.6. Composite Beads’ Swelling Behavior

The swelling behavior of the composite beads was studied using the conventional gravimetric procedure, by immersing the dry samples for different time intervals in Millipore water with different pHs, at 25 °C. Swollen composite hydrogel beads were weighed at predetermined intervals, after wiping the excess surface liquid using filter paper. To calculate the swelling ratio, SR, the three measurements made for each sample and the mean data were used in Equation (1).

SR = (W_t_ − W_0_)/W_0_, g/g, (1)

where W_0_ and W_t_ are the weights of the samples in the dried state and in the swollen state at time t, respectively.

### 4.7. Sorption Experiments

Sorption experiments were conducted using glass bottles containing 0.1 g of the sorbents and 20 mL solution of metal ions or dyes, with an initial pH of about 6, initial concentration of metal ions of 300 mg/L and initial concentration of the dye solutions of 3 × 10^−5^ M. The glass bottles were placed on a slow-moving platform shaker (LCD Digital Linear Shaker, SK-L330-Pro, DLAB Scientific Inc., Los Angeles, CA, USA) for 5–6 h. The concentrations of metal ions in the solution, before and after sorption, were analyzed using an atomic adsorption spectrometer (AAS) (ContrAA 800 Spectrometer Analytik Jena, Jena, Germany). The concentration of dyes, before and after sorption, was determined using a UV–Vis spectrophotometer (UV–Vis SPEKOL 1300, Analytik Jena, Jena, Germany), based on the calibration curves determined at the specific wavelength of 497 nm for CR and 597 nm for DR15. The sorption capacity (SC) of the hydrogel beads was calculated with Equations (2) and (3).

SC = (Cs V)/m, mg/g(2)

where Cs = sorbed concentration of ions or dyes [g/L], V = volume of the sorption solution and m = weight of used sample [g].

SC_M_ = C_sM_/M_AS_ (3)

where Cs = sorbed concentration of ions after 360 min (mol Me/g hydrogel beads), and M = active sites (AS) of hydrogel beads (mol AS/g hydrogel beads).

## Figures and Tables

**Table 1:** Pore size analysis as area, perimeter, Feret diameter and aspect ratio (mean values ± standard deviation).

Sample	Area, µm^2^	Perimeter, µm	Feret Diameter, µm	Aspect Ratio
CS/PSgAN	25.26 ± 29.71	15.69 ± 8.87	5.59 ± 3.21	1.35 ± 1.67
CS/WSgAN	29.50 ± 26.47	18.83 ± 11.00	6.18 ± 3.98	1.80 ± 1.25
CS/RSgAN	14.03 ± 9.16	12.74 ± 4.07	4.46 ± 1.48	1.15 ± 1.46
CS/PSgAN-Ax	27.74 ± 34.35	16.89 ± 8.69	6.17 ± 3.31	1.42 ± 1.53
CS/WSgAN-Ax	34.35 ± 32.53	19.27 ± 12.53	6.85 ± 4.32	1.95 ± 1.62
CS/RSgAN-Ax	16.89 ± 9.67	14.14 ± 4.02	5.00 ± 1.43	1.26 ± 1.56
CS/PSgAx	16.28 ± 13.63	11.93 ± 5.03	4.32 ± 1.70	1.29 ± 1.18
CS/WSgAx	14.37 ± 7.13	11.40 ± 3.04	3.97 ± 1.31	1.31 ± 1.12
CS/RSgAx	15.52 ± 10.99	11.24 ± 4.48	4.03 ± 1.62	1.58 ± 1.46

**Table 2:** The C/N and C/O atomic ratios calculated and determined by EDAX for composite beads.

Sample	C/N			C/O		
	PS	WS	RS	PS	WS	RS
	Calculated					
CS/SgAN	5.06			1.41		
CS/SgAN-Ax	3.54			1.24		
	At surface/In section					
CS/SgAN	7.44/5.46	6.56/6.27	6.28/6.35	2.03/1.95	1.89/1.80	2.16/1.93
CS/SgAN-Ax	6.40/4.74	6.31/5.35	5.80/5.31	2.17/2.05	1.85/1.70	1.85/1.79
CS/SgAx	6.43/4.91	6.21/5.46	5.45/5.65	1.89/1.81	1.87/1.89	1.84/1.67

**Table 3:** Molecular dynamic composition of the simulated systems.

System	Number of Water Molecules TIP3P	Number of Starch/CS Molecules	Length of the Grafted Chain	Box Size	Simulation Time (ns)
Sg	10,914	2/-	-	70 × 70 × 70	200
SgAN	16,988	2/-	3	85 × 85 × 85	200
SgAx	16,852	2/-	3	81 × 81 × 81	200
SgAN + CS	72,345	2/1	3	130 × 130 × 130	200
SgAx + CS	72,389	2/1	3	130 × 130 × 130	200

## Supplementary Materials

The following supporting information can be downloaded at: https://www.mdpi.com/article/10.3390/gels8090549/s1, Figure S1: FTIR spectra of CS/SgAN; Figure S2: FTIR spectra of CS/SgAN-Ax; Figure S3: FTIR spectra of CS/SgAx; Table S1: FTIR spectra deconvolution detailed in the region 1800–1500 cm^−1^.
