# Supplementary material for: Hydrogel Beads of Amidoximated Starch and Chitosan as Efficient Sorbents for Inorganic and Organic Compounds
Source: Gels. 2022 Aug 30;8(9):549. doi: 10.3390/gels8090549 (PMC9498570; doi:10.3390/gels8090549)
Supplement: Supplementary file 1 [file gels-08-00549-s001.zip › gels-1889013-supplementary.pdf]

gels

Hydrogel Beads of Amidoximated Starch and Chitosan as Efficient Sorbents for Inorganic and Organic Compounds

Diana Felicia Loghin, Melinda Maria Bazarghideanu, Silvia Vasiliu, Stefania Racovita, Marius-Mihai Zaharia, Tudor Vasiliu and Marcela Mihai *

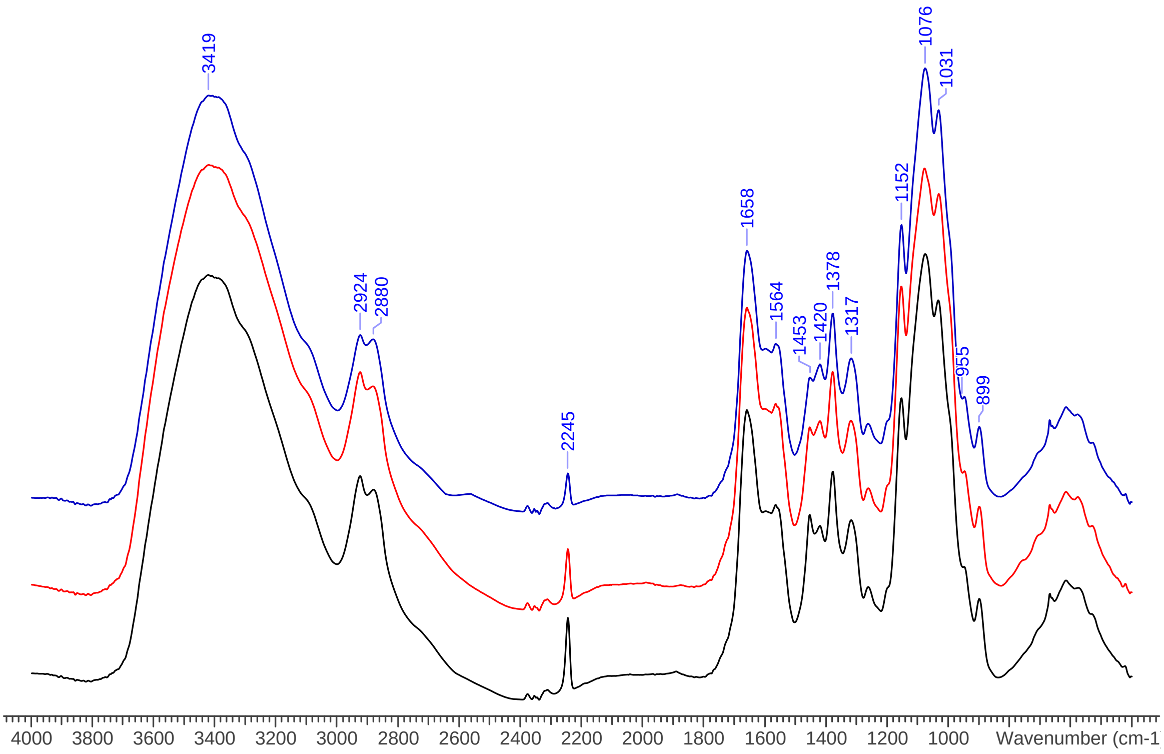

Figure S1. FTIR spectra of CS/SgAN (PS—black, WS—red, RS—blue).

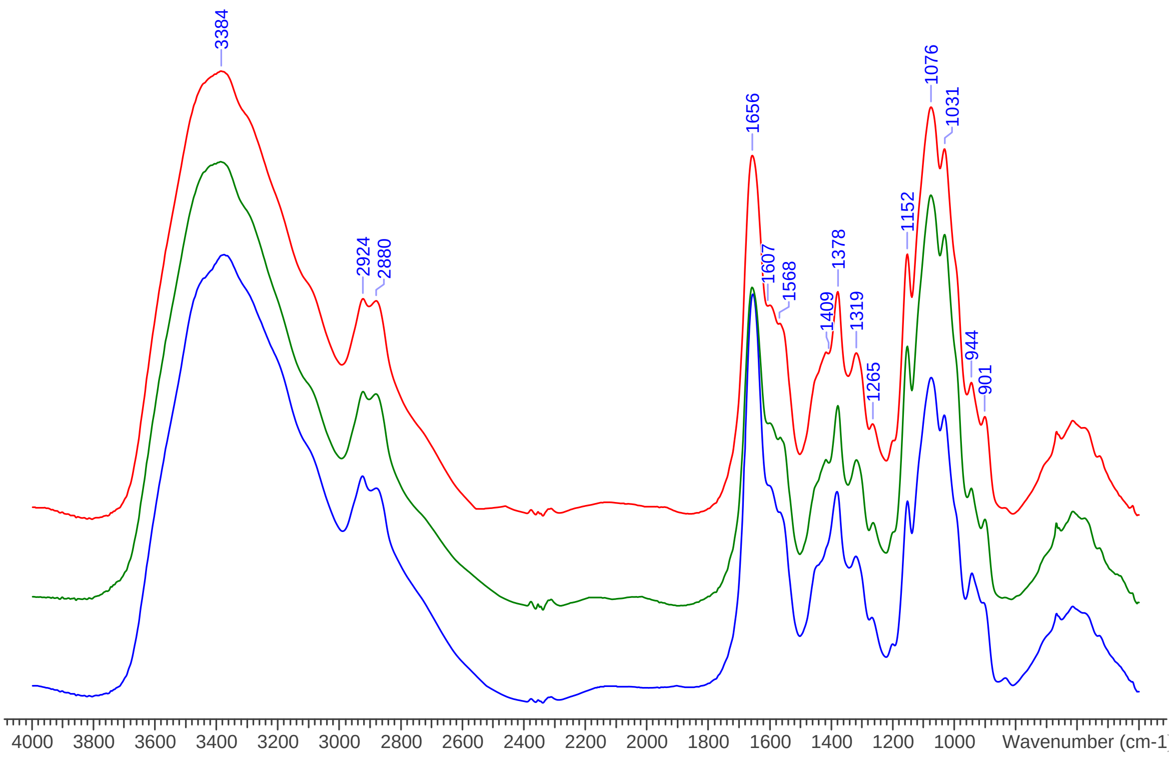

Figure S2. FTIR spectra of CS/SgAN-Ax (PS—black, WS—red, RS—blue).

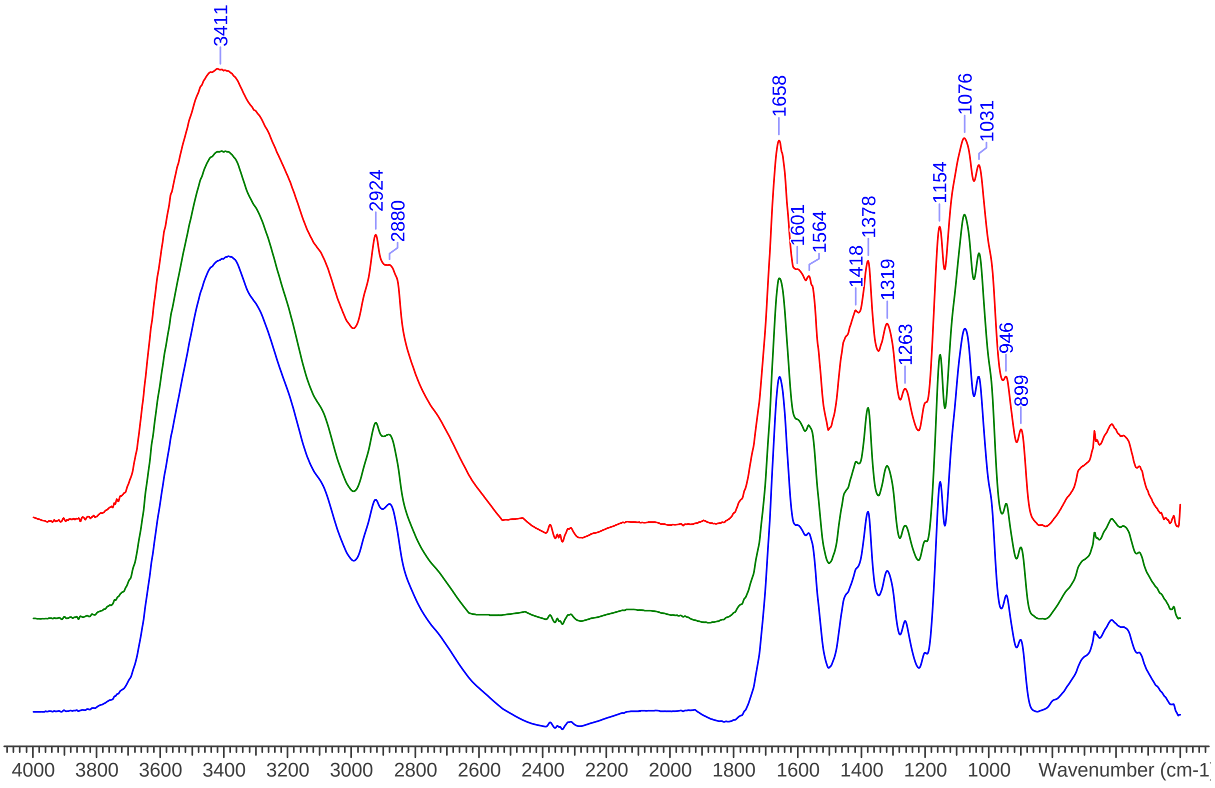

Figure S3. FTIR spectra of CS/SgAx (PS—black, WS—red, RS—blue).

Table S1. FTIR spectra deconvolution detailed in the region 1800–1500 $cm^{-1.}$

Sample	Wavelength, cm^{-1}	Peak hight	Peak area
CS/PSgAx	1732	0.01	1.2
	**1653**	**0.09**	**9.3**
	1591	0.01	0.53
	1557	0.036	2.31
CS/WSgAx	1720	0.03	2.71
	**1650**	**0.12**	**11.10**
	1595	0.01	0.47
	1559	0.05	3.59
CS/RSgAx	1733	0.02	1.1
	**1654**	**0.11**	**11.0**
	1593	0.01	0.52
	1559	0.04	2.99
CS/PSgAN-Ax	1723	0.01	0.80
	**1666**	**0.08**	**5.27**
	1633	0.03	1.21
	**1597**	**0.07**	**4.18**
	1551	0.03	1.84
CS/WSgAN-Ax	1709	0.02	1.99
	**1660**	**0.07**	**4.82**
	1632	0.01	0.41
	**1597**	**0.06**	**4.07**
	1548	0.02	1.29
CS/RSgAN-Ax	1715	0.02	1.48
	**1660**	**0.07**	**5.58**
	1629	0.02	0.97
	**1597**	**0.03**	**1.32**
	1558	0.03	2.35
